# Overcoming gaps to advance global health equity: a symposium on new directions for research

**DOI:** 10.1186/1478-4505-9-11

**Published:** 2011-02-22

**Authors:** Julio Frenk, Lincoln Chen

**Affiliations:** 1 Harvard School of Public Health, 677 Huntington Avenue, Kresge Building, 10th Floor, Boston, MA 02115-6096, USA; 2 China Medical Board, 2 Arrow Street, Cambridge, MA 02138, USA

## Abstract

The 20^th ^anniversary of the groundbreaking report of the Commission on Health Research for Development inspired a Symposium to assess progress made in strengthening essential national health research capacity in developing countries and in global research partnerships. Significant aspects of the health gains achieved in the 20^th ^century can be attributed to the advancement and translation of knowledge, and knowledge continues to occupy center stage amidst growing complexity that characterizes the global health field. The way forward will entail a reinvigoration of research-generated knowledge as a crucial ingredient for global cooperation and global health advances. To do this we will need to overcome daunting gaps, including the divides between domestic and global health, among the disciplines of research (biomedical, clinical, epidemiological, health systems), between clinical and public health approaches, public and private investments, and between knowledge gained and action implemented. Overcoming systematically these obstacles can accelerate progress towards research for equity in health and development.

## Introduction

Twenty years ago, an independent international initiative, the Commission on Health Research for Development, issued its landmark report, *Health Research: Essential Link to Equity in Development *[1], on how to "accelerate health improvements and overcome health disparities worldwide." At that time, the landscape of global health was strikingly different. There were fewer actors and severely-limited resources. The reigning paradigm was paternalistic and unidirectional: solutions were exported from the north to the south, while problems flowed from the south to be solved by the north. This seminal Report broke boldly through the crippling and rigid mindset by recommending that every country, no matter how rich or poor, should acquire key core research capability--"essential national health research" - to tackle priority national problems. Recognizing the interdependence of nations and of knowledge, the Report also underscored the value of global partnerships for health research to address commonly shared challenges.

The past 20 years have witnessed remarkable growth in the realization of the importance of global health; new avenues in global health research have opened; and research capacity in developing countries has been enhanced. In just the past decade, development assistance in health increased from US$10.7 billion in 2000 to US$21.8 billion in 2007 [2], although the fiscal impact of the recent financial crisis has yet to be registered. There are now more than 100 multilateral partnerships, agencies and initiatives in global health.

The 20^th ^anniversary of the report provided an occasion to review progress and re-launch a movement around research as a crucial ingredient for the renewal of global health cooperation. More than 200 panelists and participants from around the world convened in Boston at a Symposium hosted by the Harvard School of Public Health on May 13, 2010, to grapple with "New Directions for Global Health Research." The discussion focused on the Commission's major recommendations, which remain as valid today as 20 years ago:

1. strengthening "essential national health research" so every country can pursue research to advance national health goals;

2. mobilizing global health research partnerships so that scientists in all countries can join together in advancing knowledge as an international public good.

## Strengthening national health research systems

There is an urgent need to upgrade research capacities in all countries, especially low- and middle-income countries. This need is validated by the well documented power of knowledge to advance health through strengthening new tools, better diagnosis of health problems, guidance of health care systems, and understanding of the social determinants of health. But how should countries of all income levels strengthen their health research systems?

Capacity building is obviously crucial in countries where research infrastructure may be weak. Even the poorest nations must be capacitated to generate knowledge and be able to uptake knowledge created as a global public good and adapt it to local circumstances. Capacity building efforts are focused mostly on the development of scientific human resources in order to generate a critical mass of health researchers. It also requires the establishment of institutions capable of nurturing those researchers and their work.

Capacity strengthening support is also critical for the expansion and diversification of existing research infrastructure. This kind of support is usually provided to scientists and institutions in developing countries that are already involved in research activities, and its purpose is to enhance the research environment through construction of appropriate facilities for research, financial support to projects, access to scientific literature, partnering arrangements with stronger institutions, and creation of stable career paths.

In an opening panel chaired by Professor Adetokunbo Lucas of Nigeria, four Symposium panelists cited many examples of accelerating capacity development for essential national health research around the world. Professor Jo Ivey Boufford of the New York Academy of Medicine described the multi-country support offered by the Council on Health Research for Development (COHRED), a Geneva-based initiative, to strengthen essential national health research in the world's poorest countries. Professor Marian Jacobs of the University of Cape Town described the growing capacity for research in South Africa and welcomed the recently-announced US Government initiative (MEPI: Medical Education Partnership Initiative) aimed at capacity building of medical schools in Africa. MEPI's transferring control over the capacity agenda to African institutions was applauded. Professor Srinath Reddy, President of the Public Health Foundation of India, elaborated on the unprecedented Indian effort to establish more than half a dozen public health schools. Dr. Abdul Ghaffar of the Alliance for Health Policy and Systems Research described many collaborations among developing country scientists that foster mutual learning and expand the scope and depth of collective research endeavors.

All underscored the imperative that knowledge be translated into evidence that can guide policy and implementation. The focus should be not only on the supply side, doing more research, but also on the demand side. Panelists stressed the need to develop core competencies so that policy-makers and practitioners can be effective users of research. Leadership demands fundamental skills to be able to interpret and apply knowledge. Knowledge and ideas, in the hands of those who have the capacity to implement health policies, have enormous power to advance health.

## Establishing global health research partnerships

In addition to strengthening national health research systems, there is a concurrent need to strengthen global coordination and promotion mechanisms. The global health research system, both public and private, has grown greatly in recent years, generating new knowledge, developing new technologies, and transforming health advances. There has been proliferation of public-private partnerships, and many have brought focus to challenging problems. But how successful have they been? Can a poorer country be a genuine partner in these endeavors given its severe human and financial resource constraints? Perhaps the global architecture in partnerships can be improved to promote knowledge generation, translation and utilization.

Chaired by Professor Harvey Fineberg, President of the US Institute of Medicine, four Symposium panelists assessed the state of global health research partnerships. Dr. Tim Evans, then at the World Health Organization, described a new initiative to energize health systems research as a global public good that would be promoted by a major international conference in November 2010 in Montreux, and Dr. Anthony Mbewu, Executive Director of the Global Health Research Forum, noted that positive gains have been fostered through a steady series of similar global conferences in collaborative research. As research and knowledge are cumulative processes, such global gatherings have been useful in establishing benchmarks of progress, pointing to future priorities, and facilitating cooperation among different actors. Professor Dyann Wirth of the Harvard School of Public Health discussed several major collaborations in global research, citing specifically the Medicine for Malaria Venture. Dr. Roger Glass, Director of the US National Institute of Health Fogarty Center, reaffirmed the major US Government initiative, MEPI, financed by PEPFAR and partner agencies, to build medical school capacity in key sub-Saharan African countries.

## Overcoming gaps: symposium reflections

Future progress will depend upon overcoming divisive gaps that must be bridged systematically. Integration is the way forward. For one, there has been a gap between global and local interests, which in reality depend on each other. Global is no longer the opposite of domestic. A global outlook means that we recognize that the local and the global are united, increasingly interdependent and interconnected. We recognize that what happens in each country affects every other country.

For evidence of this we have only to look at the global economic crisis. Our world needs to have healthy children and healthy adults to be able to produce economically. This benefits all economies. Look too at pandemics: if there is an outbreak in one part of the world, it can paralyze commerce and it can eventually spread to all. We cannot ignore problems related to HIV/AIDS, tuberculosis, and malaria in poor countries without understanding that they can eventually have an impact on well-being in the wealthiest parts of the world. We cannot have a secure planet if we do not combat the root causes of insecurity, the most dramatic of which are avoidable illness, suffering, and death.

There have also been gaps among types of research: from biomedicine to clinical to epidemiological to public health and to health systems. We need to build bridges across disciplines, moving beyond the traditional silos to interdisciplinary research and education, and perhaps more importantly, across levels of analysis, upward from the gene to the entire species. The most exciting junctures in science are concentrated at the interfaces of traditional disciplines, as exemplified by genomics and bioinformatics. We are in the midst of a revolution in systems thinking which can allow us to comprehend and transform complexity.

We have also experienced very divisive gaps between public and private. Towards the end of the 20^th ^century, private initiatives accelerated, both non-profit and for-profit. The sole domination by government began to give way to many new public-private partnerships. These partnerships bring together different expertise and capacity, organizations and institutions, and sources of funding to achieve commonly shared goals. While innovative, many partnerships certainly suffer from imbalances, including the disappointing financial participation of private commercial sector in the co-funding of joint ventures. Vibrant partnerships have been created to accelerate product development of new vaccines, drugs, and diagnostics. So too have partnerships brought together private and public stakeholders in addressing priority challenges, best exemplified by the UN Millennium Development Goals. At the country level, many initiatives entail Government working with and through private agencies or private initiatives supported by public policies and funding.

Knowledge is one of the most potent instruments we have for improving health worldwide. Yet, large gaps persist between knowledge and action. This is in fact an artificial divide, much like the old traditional divide between body and mind. All knowledge shapes and inspires action, since "knowing" is also "doing." Such individual connectivity, however, may not be automatically translated institutionally in the real world.

There is a cycle of knowledge: it is produced through research; it is re-produced through education and training; it is translated into actions that improve health; and it is evaluated scientifically, which feeds back into the production of more knowledge. To improve health globally requires stoking the fires of knowledge production and ensuring that the translation into solutions is accelerated. Knowledge translation occurs in three important ways: in developing new technologies, such as vaccines, drugs and diagnostic methods; in individuals who internalize knowledge in their everyday behaviors; and in providing evidence on which to base health policy. To improve the health of populations will require not only development of specific technologies, but also of policy innovations based on scientifically-derived evidence.

Our challenge too in building bridges across these gaps is to reconcile two fundamental values: excellence and relevance [3]. This has been an artificial divide as well. While some consider these values conflicting, they are actually the same because excellence must be relevant and relevance means excellence. We need leaders, enlightened practice and policy making to provide a critical voice that is so central to the progress of societies.

## Conclusion

We have seen what knowledge-based public health action has achieved in the 20th century. Life expectancy has more than doubled. Smallpox has been wiped out, and polio is close to eradication. In HIV/AIDS we faced one of the largest public health challenges in the history of humankind, and there was a worldwide response, thanks to which four million people are on anti-retroviral treatment today. Nearly everyone thought this was impossible, not decades ago, but five years ago.

Today's challenges are huge, but we have never before had the set of resources--intellectual, technological, societal--that we do now. We have never before had the level of public awareness about the central importance of global public health. Its high visibility has been translated into enormous growth in financial assistance to health and international cooperation of an unprecedented scale.

There is a genuine sense of excitement about what lies ahead, yet an understanding that progress hinges in part on bridging and integrating these gaps. Knowledge will continue to be the key asset to sharpen our understanding of problems and to create novel solutions. In our turbulent world, still scarred all too often by ignorance and intolerance, science remains a most powerful force for enlightened social transformation.

No individual country--no matter how well endowed with human and financial resources--can generate on its own an effective response to the most pressing health challenges of our times. All nations must participate in the advancement and sharing of research-generated knowledge, along with developing the capacity to not just *adopt *the evidence, but to *adapt *it to local circumstances.

We are now ideally situated to expand global networks of collaboration and develop consortia of national and regional centers of excellence. With the legacy of creativity, inspiration, and vision brought forth by the Commission on Health Research for Development, we can continue to move forward in the cause of equity in development through research. Our outlook and our efforts must be global--we do not look inward, but rather outward, and to the future.

## Competing interests

The authors declare that they have no competing interests.

## Authors' contributions

Both authors wrote, revised and approved the manuscript.
